# Balloon Eustachian tuboplasty for obstructive Eustachian tube dysfunction: retrospective multicentre cohort study of 248 patients

**DOI:** 10.1007/s00405-023-07906-0

**Published:** 2023-03-28

**Authors:** Marta Sandoval, Juan-J Navarro, Paz Martínez-Beneyto, Mayte Herrera, Jorge Alfaro, Felipe López, Jaime Marco, Guillermo Plaza

**Affiliations:** 1 Department of Otolaryngology, Hospital Clínic Barcelona, Universitat de Barcelona (UB), C. Villarroel 170, 08036 Barcelona, Spain; 2 Departament de Medicina i Especialitats Médicoquirúrgiques, Facultat de Medicina, Universitat de Barcelona (UB), C. Casanova 143, 08036 Barcelona, Spain; 3 Department of Otolaryngology, Hospital Universitario de Donostia, Donostia-San Sebastian, Spain; 4 Department of Otolaryngology, Hospital Clínico Universitario, Valencia, Spain; 5 Department of Otolaryngology, Hospital Universitario de Fuenlabrada, Universidad Rey Juan Carlos, Madrid, Spain; 6 Department of Otolaryngology, Hospital Quirónsalud Zaragoza, Zaragoza, Spain; 7 Department of Otolaryngology, Hospital Sant Joan Despí-Moisès Broggi, Barcelona, Spain

**Keywords:** Balloon eustachian tuboplasty (BET), Eustachian tube dysfunction, Valsalva manoeuvre, Chronic serous otitis media (CSOM), Baro-challenge

## Abstract

**Objective:**

To present the results after balloon eustachian tuboplasty (BET) in patients with obstructive Eustachian tube dysfunction (OETD) grouped up into: baro-challenge, chronic serous otitis media and adhaesive otitis media.

**Methods:**

A retrospective study was carried out on patients who underwent BET surgery. As outcome measures, otoscopy, tympanometry, Eustachian tube dysfunction questionnaire-7 (ETDQ-7) and ability to perform the Valsalva manoeuvre were recorded at baseline and at 3, 12 and 24 months after BET. A *p* value of 0.05 was used to indicate a statistically significant difference for all statistical tests.

**Results:**

Three hundred and nineteen ears (248 patients) were included with a 3-month follow-up, 272 ears had a 12-month follow-up, and 171 ears had 24-month follow-up. Globally, a statistical significance improvement in all groups in all outcome measures was found. According to BET indication, in the baro-challenge group, there was no improvement in otoscopy, but ETDQ-7, Valsalva manoeuvre and tympanogram improved significantly. In the chronic serous otitis media group, otoscopy, ETDQ-7 and Valsalva manoeuvre were significantly improved in all the three timelines, including the avoidance of a new transtympanic tube after the BET in over 80% of cases. In the adhaesive otitis media group, Valsalva manoeuvre improved significantly, ETDQ-7 decreased and tympanogram improved but not significantly. Few mild complications were reported.

**Conclusions:**

BET is an effective method for the treatment of OETD in all etiologic groups. The greatest benefit was observed in patients with baro-challenge. A long-term follow-up is recommended since the benefit seems to increase over time.

**Supplementary Information:**

The online version contains supplementary material available at 10.1007/s00405-023-07906-0.

## Introduction

Obstructive Eustachian tube dysfunction (OETD) is a commonly diagnosed condition. It is considered dynamic when caused by defects in Eustachian tube (ET) muscular function, and anatomic when caused by blockage of the ET. When there is no explanation for a dynamic or anatomical cause, the condition is classified as functional, usually related to mucosa thickening due to upper airway inflammation [1, 2]. Baro-challenge is a subclinical variant of OETD in which the symptoms present only under conditions of atmospheric pressure changes (for example scuba diving or flying) [3].

The systematic review and meta-analysis of treatment outcomes after balloon eustachian tuboplasty (BET) for OETD, published by Froehlich et al. in 2020 [4], established that BET achieves significant changes in both, subjective and objective, measurable outcomes, thus verifying the efficacy of this procedure clinically and statistically. Several systematic reviews [4–10] could also show promising results with an improvement in subjective symptoms of OETD in 73–98% of patients [6]. However, most of the studies have been conducted in non-homogeneous groups of patients and with different success criteria which makes comparison between groups difficult.

A Spanish consensus paper established a rationale for BET indications in relation to distinct pathologies contributing to tubal dysfunction [11]:

- Chronic serous otitis media (CSOM) with recurrence after two previous tympanostomy tubes (TT).
- Adhaesive otitis media, grade I or II of the Sade’s scale [12].
- Baro-challenge induced OETD, presenting ET dysfunction only when pressure changes affect the dysfunction of the ET.
- Patients with OETD scheduled to middle ear procedures (cholesteatoma surgery, revision tympanoplasty).

Despite the increasing evidence for the efficacy of BET, there are few reports showing the results in different BET indications of OETD patients, establishing different specific success criteria for each group.

The aim of this study is to present overall results of BET in the treatment of OETD and specific results according the patient’s indication from six Spanish institutions.

## Materials and methods

A retrospective chart review of all consecutive patients with a history of OETD treated by BET in six institutions from March 2014 to February 2020 was designed. Ethical approval was waived by the local Ethics Committee of the University Hospital de Fuenlabrada (APR-16-10) in view of the retrospective nature of the study and all the procedures being performed were part of the routine care.

Patients with chronic OETD with more than 3 months of evolution and lack of response to usual medical treatment (oral corticosteroids, nasal corticosteroids, decongestants, etc.) were recruited, and in the specific case of patients with CSOM, those who had been treated at least twice with a TT.

Exclusion criteria were: head and neck tumours, maxillofacial malformations, chronic rhinosinusitis with or without polyposis, previous head and neck radiotherapy, and patulous Eustachian tube.

OETD diagnosis was made on the basis of symptomatology including symptoms such as discomfort in pressure changes, frequent ear crackling or popping, hearing loss and autophony. The Spanish validation of the Eustachian tube dysfunction questionnaire-7 (ETDQ-7) was used to record the patient’s symptoms [13].

OETD was also confirmed through suggestive findings on otomicroscopy, negative Valsalva manoeuvre and/or type B or C tympanograms. Findings in the tympanic membrane were classified as normal or abnormal. The efficacy of the Valsalva manoeuvre was validated with the patient either in supine or in a sitting position, and graded as positive or negative; the Valsalva manoeuver refers only to the patient’s ability to perform the manoeuvre strictly, the result was considered positive if there was an objective visualisation of the mobilisation of the tympanic membrane under otomicroscopic view, or a subjective assessment by the patient if he/she noticed the crackling in the ear. Patients were not asked to perform a Toynbee manoeuver afterwards. Objective testing demonstrated normal (type A), flat (type B), or negative (type C) curves on tympanometry. Improvement in tympanometry was defined as a change from a type B to type A or type C, or from a type C to a type A tympanometry.

The indication for BET was according to the Spanish Consensus on treatment of OETD [11].

The success criteria established in the consensus for each group of Eustachian tube dysfunction were:

- In patients with baro-challenge dysfunction, the absence of symptom triggered by pressure changes together with the presence of an efficient Valsalva manoeuvre.
- In patients with CSOM, significant and sustained over the time improvement in associated symptomatology, together with the absence of associated hearing loss that would justify another TT.
- In patients with tympanic retraction (according to the Sadé Grading System of atelectasis), we included only the grades I-II, the presence of an efficient Valsalva manoeuvre in order to stabilise the retraction and absence of progression in otomicroscopic controls.

All procedures were performed under general anaesthesia. In most of the patients, the BET was performed with the Tubavent^®^ balloon (Spiggle and Theis, Medizintechnik GmbH, Overath, Germany), and in few cases with the XprESS ENT Dilation System^®^ (Entellus Medical, Plymouth, MN, USA). No comparison was made between the two systems. The technique consisted in introducing the rigid endoscope and the curved inserter with the balloon catheter through the same nostril, avoiding any mucosal damage to prevent bleeding. Once inserted in the Eustachian tube, the catheter was displaced through the canal of the inserter and fitted carefully into it, without any resistance. Then the manometer was used to inflate the balloon to a pressure of 10 bar, which was maintained for 2 min. After, the balloon was withdrawn, either deflated or inflated, depending upon the preferences of the surgeon.

### Statistical analysis

Statistical analysis was performed with RStudio and R, version 4.1.2 [14, 15]. A descriptive analysis included frequency and proportions that were derived from categorical variables and mean and standard deviations for numerical variables. Ninety-five percentage of confidence interval (CI) was calculated for proportions and means. Analysis of continuous ETDQ-7 measures (comparison of pre- and post-treatment means) was performed by means of the paired *t*-test. For this study, the null hypothesis was that there was no difference between pre- and post-treatment with respect to ETDQ-7. In addition, an analysis of proportions was done for the improvement in otomicroscopy, Valsalva manoeuvre and tympanometry. Comparisons between pre- and post-treatment proportions were done by means of the exact McNemar test for matched categorical variables. The null hypothesis for this case was the same, no difference between pre- and post-treatment classification. A *p* value of 0.05 was used to indicate a statistically significant difference for all statistical tests.

## Results

Altogether, 284 patients (371 ears) were treated by BET. Twenty-seven patients were associated with a tympanoplasty and were, therefore, excluded to avoid potential confounding factors.

**Fig. 1:**
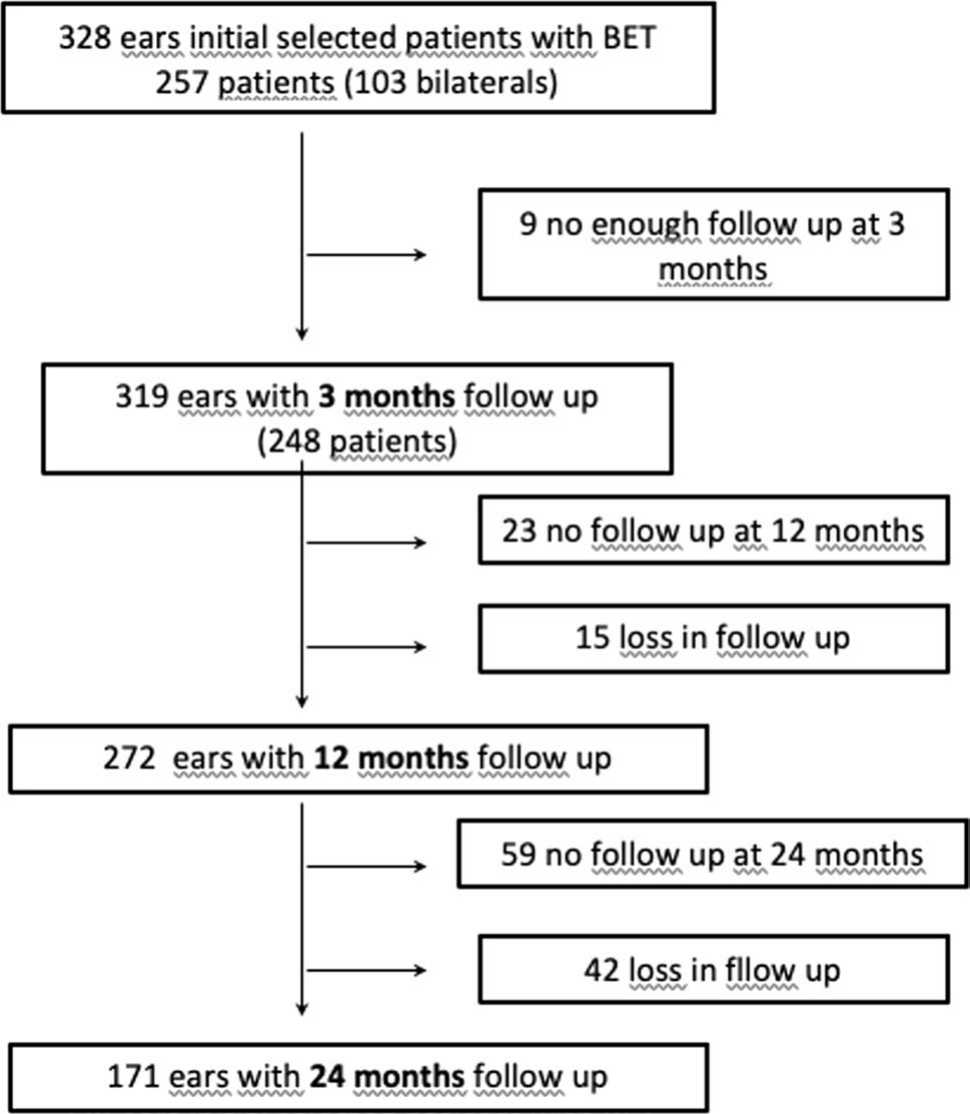
Flow of patients, from first recruitment to each timelapse follow-up

A total of nine patients were excluded for lost to follow-up. Thus, 248 patients (319 ears) were controlled after BET, with a minimum follow-up of 3 months, 272 ears (207 patients) had a 12 months of follow-up, and 171 (130 patients) had a 24 months of follow-up after the procedure. The mean follow-up was 17.16 months (Fig. 1).

One-hundred and fifty-one patients (62.69%) were males, and 90 (37.31%) were females, with a mean age of 41.29 years (range 4–79 years). One-hundred and sixty-nine were right (52.98%) and 150 (47.02%) were left ears, and 71 patients (28.62%) underwent a bilateral BET.

### Global results

Table 1 summarises the findings of the whole series in otomicroscopy, ETDQ-7, and performance of Valsalva manoeuvre after 3, 12 and 24 months, as compared to the pre-treatment results. All outcome measures significantly improved. The results obtained in the different tympanogram groups at the postoperative control times classified as A, B and C were compared. All results were statistically highly significant showing a tympanogram improvement (Table 1). The percentage of ears improving tympanogram in all groups from baseline to 3 months is 63.73% (95% CI 55.23–72.23), 79.66% at 12 months (95% 73.02–86.3) and 78.45% (95% CI 70.00–86.90) at 2 years.

**Table 1:** Results in all groups

Outcome	Time points	Otoscopy			ETDQ-7			Valsalva manoeuver			Objective success		
		Difference	IC95dif	*p* values	Difference	IC95dif	*p* values	Difference	IC95dif	*p* values		Yes	No
Normal	3 m To PRE	43.29	33.12–53.46	** < 0.0001**	− 7.98	− 10.09 to − 6	** < 0.0001**	56.91	45.53–68.29	** < 0.0001**	**3 m (IC 95)**	268 (86.45%) (82.35–90.55)	42 (13.55%) (3.2–23.9)
Normal	12 m To PRE	46.61	36.49–56.73	** < 0.0001**	− 10.87	− 12.72 to − 9	** < 0.0001**	67.23	56.44–78.02	** < 0.0001**	**12 m. (IC 95)**	232 (85.29%) (80.73–89.85)	40 (14.71%) (3.73–25.69)
Normal	24 m To PRE	45.55	34.62–56.48	** < 0.0001**	− 12.72	− 14.57 to − 11	** < 0.0001**	66.66	55.33–77.99	** < 0.0001**	**24 m (IC 95)**	155 (90.64%) (86.05–95.23)	22(12.87%) (− 4.91–23.63)

Tympanogram				
Outcome	Time points	Difference	IC95dif	*p* values
A	3 m to PRE	46.46	33.9 to 59.02	** < 0.0001**
A	12 m to PRE	58.59	46.65 to 70.53	** < 0.0001**
A	24 m to PRE	58.51	45.74 to 71.28	** < 0.0001**
B	3 m to PRE	− 24.55	− 47.59 to − 10.74	5e–04
B	12 m to PRE	− 33.78	− 48.66 to − 18.9	** < 0.0001**
B	24 m to PRE	− 32.71	− 50.05 to − 15.37	2e–04
C	3 m to PRE	− 21.90	− 41.01 to − 6.7	0.0047
C	12 m to PRE	− 24.82	− 40.73 to − 8.91	0.0022
C	24 m to PRE	− 25.81	− 44.36 to − 7.26	0.0064

The outcomes are shown comparing each one of the determinations from the baseline to each one of the time points studied

*PRE* preoperatively, *IC95dif* 95% interval confidence difference, *ETDQ-7* Eustachian tube dysfunction questionnaire-7. In bold those statisticall significant results

### Results by group according to bet indication

This section shows the results obtained in the different three groups into which the patients were divided according to the reason for the indication of BET: baro-challenge, CSOM or adhaesive otitis media grade I or II.

#### Baro-challenge induced ETD

Out of the 319 ears treated by BET, 107 (33.55%) belonged to this group. The proportion of ears without pathological findings on otoscopy (normal ear drum) did not decrease significantly. ETDQ-7 mean scores decreased significantly from the initial value for all follow-up periods. The patients’ ability to achieve an efficient Valsalva manoeuvre also improved significantly in all postoperative controls (Table 2). The baro-challenge condition is defined according to the Spanish Consensus on OETD (11) when patients report a sensation of discomfort and pain from pressure in the ears, particularly with changes in atmospheric pressure (dysbarism), either in aviation or diving, although there is no specific registry for each of these conditions, nor whether the patients affected by baro-challenge were aviation or diving professionals or not. Similarly, the results obtained in the different tympanogram groups at the different postoperative control times classified as A, B and C were compared (Table 2). The percentage of ears with improving tympanogram in the baro-challenge group from baseline to 3 months was 86.27% (95% CI 76.1–96.44), 91.84% at 12 months (95% CI 83.84–99.84) and 87.80% (95% CI 77.11–98.49) at 2 years, showing long-term and stable good results.

**Table 2:** Results in the baro-challenge group

Outcome	Time Points	Otoscopy			ETDQ-7			Valsalva manoeuver			Objective success		
		Difference	IC95dif	*p* values	Difference	IC95dif	*p* values	Difference	IC95dif	*p* values		Yes	No
Normal	3 m. To PRE	− 0.96	− 2.84 to 0.92	0.3177	− 9.05	− 13.95 to − 4	3e − 04	54.43	37.35–71.51	** < 0.0001**	**3 m (IC 95)**	91 (87.5%) (80.7–94.3)	13 (12.5%) (− 5.48 to 30.48)
Normal	12 m. To PRE	− 5.43	− 10.19 to − 0.67	0.0254	− 16.36	− 20.59 to − 12	** < 0.0001**	60.99	44.6–77.38	** < 0.0001**	**12 m (IC 95)**	84 (91.3%) (85.27–97.33)	8 (8.7%) (− 10.83 to 28.23)
Normal	24 m. To PRE	− 5.00	− 10.66 to 0.66	0.0833	− 18.49	− 22.44 to − 15	** < 0.0001**	61.31	44.56–78.06	** < 0.0001**	**24 m (IC 95)**	60 (100%) (100–100)	0 (0.0%) (NAN–NAN)

	Normal	Abnormal	N (media)	SD	Normal	Abnormal
Baseline (IC 95)	107 (100%) (100–100)	0 (0%) NaN-NaN	29 (28.41)	10.28 (24.67–32.15)	35 (33.33%) (17.71–48.95)	70 (66.67%) (55.63 to 77.7)
3 months (IC 95)	103 (99.04%) (97.16–100.92)	1 (0.96%) (− 18.15 to 20.07)	28 (19.36)	8.53 (16.2–22.52)	86 (87.76%) (80.83–94.69)	12 (12.24%) (− 6.3 to 30.78)
12 months (IC 95)	87 (94.57%) (89.81–99.33)	5 (5.43%) (− 14.43 to 25.29)	22 (12.05)	4.71 (10.08–14.02)	83 (94.32%) (89.34–99.3)	5 (5.68%) (− 14.61 to 25.97)
24 months (IC 95)	57 (95%) (89.34–100.66)	3 (5%) (− 19.66 to 29.66))	12 (9.92)	2.23 (8.66–11.18)	53 (94.64%) (88.58–100.7)	3 (5.36%) (− 20.13 to 30.85)

Tympanogram				
Outcome	Time points	Difference	IC95dif	*p* values
A	3 m to PRE	50.28	34.2 to 66.36	** < 0.0001**
A	12 m to PRE	53.09	37.29 to 68.89	** < 0.0001**
A	24 m to PRE	49.16	32.19 to 66.13	** < 0.0001**
B	3 m to PRE	− 5.77	− 32.45 to 22.42	0.6882
B	12 m to PRE	− 4.26	− 33.47 to 24.95	0.775
B	24 m to PRE	− 3.23	− 35.45 to 28.99	0.8442
C	3 m to PRE	− 44.51	− 70.43 to − 20.01	4e − 04
C	12 m to PRE	− 48.82	− 74.97 to − 22.67	3e − 04
C	24 m to PRE	− 45.93	− 75.2 to − 16.66	0.0021

The outcomes are shown comparing each one of the determinations from the baseline to each one of the time points studied

*PRE* preoperatively, *IC95dif* 95% interval confidence difference, *ETDQ-7* Eustachian tube dysfunction questionnaire-7. In bold those statisticall significant results

#### Chronic serous otitis media (CSOM)

This group includes 169 out of the 319 (52.97%) ears. Thirty-two (18.9%) were equal to or younger than 14 years (mean age 9.3 years), up to which the Spanish health system considers the paediatric age. Only two children underwent bilateral BET, and in the rest, the procedure was performed unilaterally. The proportion of ears with altered otoscopy decreased significantly along all follow-up periods. ETDQ-T mean scores in CSOM group also decreased significantly. The patient’s ability to achieve an efficient Valsalva manoeuvre in CSOM group improved significantly in all postoperative controls. An objective improvement was found at all cutoff periods. The difference in tympanogram type was also significant in type A and B, but did not reach the statistical significance in type C (Table 3).

**Table 3:** Results in the chronic serous otitis media (CSOM) group

Outcome	Time points	Otoscopy			ETDQ-7			Valsalva manoeuver			Objective success		
		Difference^1^	IC95dif	*p* values	Difference^1^	IC95dif	*p* values	Difference^1^	IC95dif	*p* values		Yes	No
Normal	3 m to PRE	67.64	50.78–84.5	** < 0.0001**	− 7.87	− 9.92 to − 6	** < 0.0001**	54.88	38.45–71.31	** < 0.0001**	**3 m (IC 95)**	139 (85.28%) (79.39–91.17)	24 (14.72%) (0.54–28.9)
Normal	12 m to PRE	76.68	60.3–93.06	** < 0.0001**	− 9.04	− 10.94 to − 7	** < 0.0001**	68.03	52.65–83.41	** < 0.0001**	**12 m (IC 95)**	124 (84.93%) (78.63–91.23)	22 (15.07%) (0.12–30.02)
Normal	24 m to PRE	73.91	56–91.82	** < 0.0001**	− 10.62	− 12.64 to − 9	** < 0.0001**	63.84	46.76–80.92	** < 0.0001**	**24 m (IC 95)**	72 (87.80%) (80.24–95.36)	10 (12.20%) (− 8.09–32.49)

	Normal	Abnormal	N (media)	SD	Normal	Abnormal
Baseline (IC 95)	7 (4.14%) (− 10.62–18.9)	162 (95.86%) (92.79–98.93)	75 (21.39)	7.32 (19.73–23.05)	29 (18.59%) (4.43–32.75)	127 (81.41%) (74.64–88.18)
3 months (IC 95)	117 (71.78%) (63.62–79.94)	46 (28.22%) (15.21–41.23)	77 (13.52)	5.42 (12.31–14.73)	108 (73.47%) (65.14–81.8)	39 (26.53%) (12.67–40.39)
12 months (IC 95)	118 (80.82) (73.72–87.92)	28 (19.18) (4.6–33.76)	63 (12.35)	3.75 (11.42–13.28)	123 (86.62%) (80.6–92.64)	19 (13.38%) (− 1.93–28.69)
24 months (IC 95)	64 (78.05) (67.91–88.19)	18 (21.95%) (2.83–41.07)	26 (10.77)	3.01 (9.61–11.93)	61 (82.43%) (72.88–91.98)	13 (17.57%) (− 3.12–38.26)

Tympanogram				
Outcome	Time points	Difference	IC95dif	*p* values
A	3 m to PRE	43.15	24.22 to 62.08	** < 0.0001**
A	12 m to PRE	62.18	44.59 to 79.77	** < 0.0001**
A	24 m to PRE	64.48	45.24–83.72	** < 0.0001**
B	3 m to PRE	− 36.17	− 70.15 to − 20.48	** < 0.0001**
B	12 m to PRE	− 54.46	− 72.24 to − 36.68	** < 0.0001**
B	24 m to PRE	− 54.17	− 76.87 to − 31.47	** < 0.0001**
C	3 m to PRE	− 6.99	− 31.41 to 14.1	0.516
C	12 m to PRE	− 7.73	− 29.31 to 13.85	0.4826
C	24 m to PRE	− 10.32	− 36.77 to 16.13	0.4444

The outcomes are shown comparing each one of the determinations from the baseline to each one of the time points studied

*PRE* preoperatively, *IC95dif* 95% interval confidence difference, *ETDQ-7* Eustachian tube dysfunction questionnaire-7. In bold those statisticall significant results

The percentage of ears improving tympanogram in the CSOM group from baseline to 3 months was 54.55% (95% CI 42.54–66.56), 75.44% at 12 months (95% CI 66.34–84.54) and 74.19% (95% CI 61.54–86.84) at 2 years.

The criterion for considering BET successful in patients with CSOM was a significant improvement in associated symptomatology (ETDQ-7) together with the absence of associated hearing loss that would justify the need for repositioning a TT. The success rate at 3 months was 85.28% (95% CI 80.07–94.3), at 12 months, it was 84.93% (95% CI 85.27–97.33), and at 2 years, it was 87.80% (95% CI 80.24–95.36) (Table 3).

#### Adhaesive otitis media

This group was composed of 43 (13.48%) with tympanic retraction with the Sadé’s grades I–II. None of the ears of this group had normal otomicroscopy at baseline. The proportion of ears with normal otoscopy significantly increased in all follow-up period. ETDQ-7 mean scores in this group decreased, but not significantly. None of the 43 patients in this group was able to preoperatively achieve a positive Valsalva and this changed significantly in all postoperative controls. The objective success achieved at 3 months (88.77%) decreased slightly at 12 months and levelled off at 2 years of follow-up. No statistical significance was found in the tympanogram in any follow-up period (Table 4).

**Table 4:** Results in the adhaesive otitis media group

Outcome	Time points	Otoscopy			ETDQ-7			Valsalva maneuvre			Objective success		
		Difference^1^	IC95dif	*p* values	Difference	IC95dif	*p* values	Difference	IC95dif	*p* values		Yes	No
Normal	3 m to PRE	58.14	NaN–NaN	** < 0.0001**	− 4.24	− 14.9 to 6	0.4358	73.70	38.11–109.29	** < 0.0001**	**3 m (IC 95)**	38 (88.37%) (78.18–98.56)	5 (11.63%) (− 16.47–39.73)
Normal	12 m to PRE	55.88	NaN–NaN	** < 0.0001**	− 7.62	− 17.09 to 2	0.1148	83.58	49.21–117.95	** < 0.0001**	**12 m (IC 95)**	24 (70.59%) (52.36–88.82)	10 (29.41%) (1.17–57.65)
Normal	24 m to PRE	62.07	NaN–NaN	** < 0.0001**	− 11.12	− 19.19 to − 3	0.0069	90.12	56.94–123.3	** < 0.0001**	**24 m (IC 95)**	23 (79.31%) (62.75–95.87)	6 (20.69%) (− 11.72–53.1)

	Normal	Abnormal	N (media)	SD	Normal	Abnormal
Baseline (IC 95)	0 (0%) (NaN–NaN)	43 (100%) (100–100)	8 (21.62)	10.78 (14.15–20.09)	2 (5.71%) (− 26.45–37.87)	33 (94.29%) (86.37–102.21)
3 months (IC 95)	25 (58.14%) (38.8–77.48)	18 (41.86%) (19.07–64.65)	8 (17.38)	10.98 (9.77–24.99)	27 (79.41%) (64.16–94.6)	7 (20.59%) (− 9.37–50.55)
12 months (IC 95)	19 (55.86%) (33.55–78.21)	15 (44.12%) (18.99–69.25)	4 (14.00)	5.94 (8.18–19.52)	25 (89.29%) (77.17–101.41)	3 (10.71%) (− 24.28–45.7)
24 months (IC 95)	18 (62.07%) (39.65–84.49)	11 (37.93%) (9.26–66.6)	4 (10.50)	3.11 (7.45–13.55)	23 (95.83%) (87.66–104)	1 (4.17%) (− 35.01–43.35)

Tympanogram				
Outcome	Time points	Difference	IC95dif	*p* values
A	3 m to PRE	48.21	6.06—90.36	0.025
A	12 m to PRE	57.93	15.87—99.99	0.0069
A	24 m to PRE	52.38	8.93—95.83	0.0181
B	3 m to PRE	− 21.43	-62.64—22.56	0.3397
B	12 m to PRE	− 18.65	-66.36—29.06	0.4436
B	24 m to PRE	− 13.10	-59.55—33.35	0.5804
C	3 m to PRE	− 26.79	-86.55—20.47	0.2666
C	12 m to PRE	− 39.29	NaN—NaN	NaN
C	24 m to PRE	− 39.29	NaN—NaN	NaN

The outcomes are shown comparing each one of the determinations from the baseline to each one of the time points studied

*PRE* preoperatively, *IC95dif* 95% interval confidence difference, *ETDQ-7* Eustachian tube dysfunction questionnaire-7. In bold those statisticall significant results

The percentage of ears improving tympanogram in the adhaesive otitis media group from baseline to 3 months is 61.90% (95% CI 35.5–88.3), 71.43% at 12 months (95% CI 43.43–99.43) and 69.33% (95% CI 39.08–99.38) at 2 years.

### Complications

A total of nine patients (3.4%) had mild or moderate complications. A case of subcutaneous emphysema in the upper hemithorax in a patient who performed the Valsalva manoeuvre very intensely, resolved within a few days. Antibiotics were administered prophylactically; Valsalva manoeuvre was prohibited until the emphysema resolved. After bilateral tubal dilation, a patient presented with a unilateral intratympanic hematoma in the antero-inferior quadrant, that resolved spontaneously. Another patient had mild otorrhagia with a small unilateral tympanic perforation, possibly because of a barotrauma during dilation, which both resolved spontaneously. Four patients presented mild epistaxis, of which only one case required nasal packing for a few hours, and lastly, one patient presented mild vertigo after the procedure, and was recommended not to perform the Valsalva manoeuvre until after one week. None of these cases had presented incidents during the dilation.

## Discussion

Since its introduction as a treatment of OETD [16, 17], there is enough scientific evidence of the BET benefits. BET has shown to be safe and superior to drug treatment, be, presenting few side effects [18, 19] and stable long-term results [10, 20]. There are several systematic reviews [4, 7–9, 21] and a recent meta-analysis of 12 studies, concluding that BET is associated with improvement in subjective and objective treatment outcomes and that its results are stable at 12 months after dilation [4].

OETD comprises a range of different conditions that can be evolutionary, such as baro-challenge, CSOM, adhaesive otitis media or cholesteatoma [2]. As there are no standardised evaluation protocols to assess BET outcomes, almost all publications use the same parameters (ETDQ-7, otomicroscopy, tympanometry and Valsalva manoeuvre) to evaluate its results, regardless of the pathology derived from the tubal dysfunction. Due to the different conditions and non-homogeneous groups of patients, it is difficult to compare the results among publications.

The parameters to evaluate the success are used indistinctly with the different pathologies causing OETD. For example, a patient with CSOM with previous several TTs may be considered a failure if the tympanometry and the Valsalva manoeuvre are not improved after the surgery. However, if the case has been resolved avoiding the need for future TT and achieving normal and stable hearing results, it should be accepted as a success.

We believe that each indication for BET deserves a different monitoring and outcome evaluation. Available objective measurement tests do not correlate well with the patient’s symptoms [22, 23]. In addition, the validity of the classical tympanometry values to assess tubal dysfunction has recently been questioned [24, 25].

With regards to this statement, there are not many publications analysing the results in the different groups of OETD. Few of them evaluated the results of BET in patients with CSOM [26–30], and some other in baro-challenge [31–33], including a systematic review [34]. Finally, to the best of our knowledge, there are not literature reports about BET in adhaesive otitis media as stand-alone treatment nor associated with other procedures such as cartilage tympanoplasty [35].

Following the consensus paper of the Spanish ENT Society [11], our best results were obtained in the baro-challenge group with an effectiveness of 87.5%, 91% and 95% at 3, 12 and 24 months, respectively. The success criteria were the absence of symptoms triggered by atmospheric pressure changes, together with the presence of a positive Valsalva manoeuvre. These results improved over the time, as Utz et al. [31] seen on nine patients. Ungar et al. [33] and Cheng et al. [36] published a 100% success rate in this group of patients. A recent systematic review concludes that BET appears to be effective in improving symptoms in baro-challenge induced Eustachian dysfunction [37]. Patients with a baro-challenge ET dysfunction seem to be the best candidates for BET as their success is the highest. Many of them had normal tympanograms and otosmicroscopy prior to BET (50% and 100%, respectively). Subsequently, both are possibly not the best parameters to predict or to assess the outcome success rate. The ability to perform the Valsalva manoeuvre and the lack of symptoms during baro-challenging activities afterwards seem to be better predictors of success.

According to our results, CSOM would be the second-best indication for BET. The improvement was significant and sustained over the time with regards to the ETDQ-7, together with the absence of middle ear effusion with a conductive hearing loss that would justify the need for TT. We report an effectiveness of 85.28%, 84.93% and 87.80% at 3, 12 and 24 months, respectively. With an average success rate in this group of 85%, the recurrence rate is of 15%. The results remain stable over time and are in agreement with those published by Li et al. [30], who compared a group of CSOM patients treated by BET plus TT with a control group treated by TT alone. The success criteria were defined by Ockermann et al. [16] and the recurrence rate was of 14% at 2 years in the BET plus TT, as compared to 25% in the TT alone group. Liang et al. [27] randomised 90 patients in 3 groups: BET, BET plus myringotomy and only myringotomy. The results obtained at 6 months confirm the superiority of BET and BET plus myringotomy over myringotomy alone, both in the otoscopy (80% and 86.6%, respectively, vs. 6.7%) as well as in the increase of type A tympanometry (80% and 83.3% vs. 7%). In our cohort, the percentage of patients who normalise otoscopic findings and achieve a type A tympanogram match the groups treated with BET (80%/80%, respectively) and BET plus myringotomy (86.6%/83.3%); however, in our hands, not all patients required another TT after dilatation showed a normal tympanogram despite maintaining normal hearing. Subsequently, the tympanogram does not seem to be a fundamental criterion in the evaluation of the success of treatment in patients with CSOM, although its improvement could be a good indicator of the efficacy of BET.

Similarly, Si et al. [29] report a significantly lower recurrence rate at 12 months in patients with CSOM treated by BET (24%) and BET plus myringotomy (22%) versus those treated by myringotomy alone (64%). This recurrence rate decreases to 10% in patients undergoing simultaneous middle ear irrigation with methylprednisolone. Although myringotomy does not appear to improve long-term outcomes when performed simultaneously with BET, it may help shorten the recovery period for middle ear effusion [26, 27].

To the best of our knowledge, this is the first study presented in a group of patients with adhaesive otitis media Sade’s grade I–II treated with BET only. The objective in this group, and therefore, the success criterion, was to achieve an effective Valsalva manoeuvre to stabilise the retraction, objectively evidenced by otoendoscopy or otomicroscopy, together with the absence of progression of the retraction/adhaesion in otomicroscopic follow-up. The success rate obtained is somewhat lower than in patients with baro-challenge or with CSOM, being of 88.37%, 70.59% and 79.31% at 3, 12 and 24 months, respectively. It is worthwhile highlighting that the results fluctuate more than in the other study groups and that around 50% will normalise the tympanogram.

Only one publication specifies the results by tubal pathology groups in an Australian cohort [36]. The main difference is that the group defined as pathology related to ETD not only included cases with atelectasis/retraction, but also others with suppurative chronic otitis media and cholesteatoma. In a similar way, their best results were obtained in the baro-challenge group, followed by CSOM, with the worst results being obtained in the group of other pathologies related to ETD.

The global results in all patients considered as a single group show a significant improvement in the long term (12–24 months) of 78–79% in the tympanogram (of which only 58% normalised), normalised otoscopy in 45–46%, effective Valsalva manoeuvre in 66–67% and a decrease in ETDQ-7 to normal values (10.5–12.3) after BET. These results are in line with the overall efficacy of BET found in two meta-analyses [4, 8], and are very similar to those published in the two long-term randomised studies [18, 19] and its subsequent extensions in the long term [10, 20].

## Limitations

This is a retrospective study in which the drop-outs at 3, 12 and 24 months limit the results to a shorter number of patients than initially included in the study. Not all patients included in the cohort had each of the study parameters included in the results. We used two different balloons despite the fact that in most cases Tubavent^®^ (Spiggle & Theis, Medizintechnik GmbH, Overath, Germany) was used, but the technologic parameters of both systems are the same, so no comparative study was performed.

## Conclusions

BET is an effective method for the treatment of OETD. The percentage of improvement in all study groups has been significant. The greatest benefit was observed in patients affected by baro-challenge. BET is a safe method with few minor complications, which makes it the technique of choice in patients with OETD. A long-term follow-up is recommended since the benefit of BET seems to increase over time.

## Supplementary Information

Below is the link to the electronic supplementary material.

Supplementary file1 (PDF 395 KB)

Supplementary file2 (DOCX 20 KB)
