# Supplementary material for: Balloon Eustachian tuboplasty for obstructive Eustachian tube dysfunction: retrospective multicentre cohort study of 248 patients
Source: Eur Arch Otorhinolaryngol. 2023 Mar 28;280(9):4045–55. doi: 10.1007/s00405-023-07906-0 (PMC10382357; doi:10.1007/s00405-023-07906-0)
Supplement: Supplementary file 1 — Supplementary file1 (PDF 395 KB) [file 405_2023_7906_MOESM1_ESM.pdf]

Mrs. Cristina Puivecino Moreno, Technical Secretary of the Ethics Committee for Research with Medicines of the University Hospital of Fuenlabrada,

CERTIFIES

That this Committee at its **meeting on February 24, 2016 (02/2016)** has evaluated the documentation presented by the Researcher corresponding to the Titled Study:

"Diagnóstico de la disfunción de la trompa de eustaquio mediante tubomanometría."

"Diagnosis of eustachian tube dysfunction by tubomanometry"

IRB of the Ethics Committee for Research with Medicines of the University Hospital of Fuenlabrada: **16/10**

And consider that:

The necessary suitability requirements of the Protocol in relation to the objectives of the study are met and the foreseeable risks and discomfort for the subject are justified.

The Researcher's capacity and available means are appropriate to carry out the study.

And this Committee accepts that the study be carried out by:

Dra. María Teresa Herrera Mera
Otorhinolaryngology Department
University Hospital of Fuenlabrada (Madrid, Spain).

Fuenlabrada, November 16, 2022

Hospital Universitario de Fuenlabrada
SaludMadrid
Comité de Ética de la Investigación con Medicamentos

Signed: Dª. Cristina Puivecino Moreno
Secretaria Técnica del CEIm
