# Supplementary material for: Balloon Eustachian tuboplasty for obstructive Eustachian tube dysfunction: retrospective multicentre cohort study of 248 patients
Source: Eur Arch Otorhinolaryngol. 2023 Mar 28;280(9):4045–55. doi: 10.1007/s00405-023-07906-0 (PMC10382357; doi:10.1007/s00405-023-07906-0)
Supplement: Supplementary file 2 — Supplementary file2 (DOCX 20 KB) [file 405_2023_7906_MOESM2_ESM.docx]

| TYMPANOGRAM IMPROVEMENT | | | | | | | | | |
| --- | --- | --- | --- | --- | --- | --- | --- | --- | --- |
|  | 3 months | | | 12 months | | | 24 months | | |
|  | N | PROPORTION | CI 95 | N | PROPORTION | CI 95 | N | PROPORTION | CI 95 |
| GENERAL | | | | | | | | | |
| Improvement | 123 | 63.73% | (55.23-72.23) | 141 | 79.66% | (73.02-86.3) | 91 | 78.45% | (70-86.9) |
| No improvement | 70 | 36.27% | (25.01-47.53) | 36 | 20.34% | (7.19-33.49) | 25 | 21.55% | (5.43-37.67) |
| BARO-CHALLENGE | | | | | | | | | |
| Improvement | 44 | 86.27% | (76.1-96.44) | 45 | 91.84% | (83.84-99.84) | 36 | 87.80% | (77.11-98.49) |
| No improvement | 7 | 13.73% | (-11.77-39.23) | 4 | 8.16% | (-18.67-34.99) | 5 | 12.20% | (-16.49-40.89) |
| CRSOM | | | | | | | | | |
| Improvement | 66 | 54.55% | (42.54-66.56) | 86 | 75.44% | (66.34-84.54) | 46 | 74.19 | (61.54-86.84) |
| No improvement | 55 | 45.45% | (32.29-58.61) | 28 | 24.56% | (8.62-40.5) | 16 | 25.81% | (4.37-47.25) |
| ADHESIVE OTITIS MEDIA | | | | | | | | | |
| Improvement | 13 | 61.90% | (35.5-88.3) | 10 | 71.43% | (43.43-99.43) | 9 | 69.33% | (39.08-99.38) |
| No improvement | 8 | 38.10% | (4.45-71.75) | 4 | 28.57% | (-15.7-72.84) | 4 | 30.77% | (-14.46-76) |

| TYMPANOGRAM | | | | | | | | | | | | |
| --- | --- | --- | --- | --- | --- | --- | --- | --- | --- | --- | --- | --- |
|  | GENERAL | | | BARO-CHALLENGE | | | CSOM | | | ADHAESIVE OTITIS MEDIA | | |
|  | A | B | C | A | B | C | A | B | C | A | B | C |
| -Baseline  (IC 95)  -3 months  (IC 95)  -12 months  (IC 95)  -24 months  (IC 95) | 59 (20.21%)  (9.96-30.46)  162 (66.67%)  (59.41-73.93)  171 (78.80%)  (72.67-84.93)  111 (78.72%)  (71.11-86.33) | 139 (47.6%)  (39.3-55.9)  56 (23.05%)  (12.02-34.08)  30 (13.82%)  (1.47-26.17)  21 (14.89%)  (-0.34-30.12) | 94 (32.19%)  (22.7-41.63)  25 (10.29%)  (-1.62-22.2)  16 (7.37%)  (-5.43-20.17)  9 (6.38%)  (-9.59-22.35) | 42 (41.58%)  26.67-56.49  79 (91.86%)  85.83-97.89  71 (94.67%)  89.44-99.9  49 (90.74%)  82.62-98.86 | 7 (6.93%)  -11.88-25.74  1 (1.16%)  -19.83-22.15  2 (2.67%)  -19.67-25.01  2 (3.70%)  -22.46-29.86 | 52 (51.49%)  37.91-65.07  6 (6.98%)  -13.41-27.37  2 (2.67%)  -19.67-25.01  3 (5.56%)  -20.37-31.49 | 13 (7.98%)  -6.75-22.71  68 (51.13%)  39.25-63.01  87 (70.16%)  60.55-79.77  50 (72.46%)  60.08-84.84 | 119 (73.01%)  65.03-80.99  49 (36.84%)  23.33-50.35  23 (18.55%)  2.66-34.44  13 (18.84)  -2.42-40.1 | 31 (19.02%)  5.2-32.84  16 (12.03%)  -3.91-27.97  14 (11.29%)  -5.29-27.87  6 (8.70%)  -13.85-31.25 | 4 (14.29%)  -20.01-48.59  15 (62.50%)  38-87  13 (72.22%)  47.87-96.57  12 (66.67%)  40-93.34 | 13 (46.43%)  19.32-73.54  6 (25%)  -9.65-59.65  5 (27.78%)  -11.48-67.04  6 (33.33%)  -4.39-71.05 | 11 (39.29%)  10.43-68.15  3 (12.50%)  -24.92-49.92  0 (0%)  NaN-NaN  0 (0%)  NaN-NaN |

2. Additional material. *N*: number; *CI*: confidence interval. Diagram of the tympanogram comparative in all groups. Both the general, baro-challenge, and CSOM groups had a statistically significant improvement at 3, 12 and 24 months compared to type A tympanometry. Improvement in the type B and C (i.e., fewer patients in these groups throughout the follow-up) was also observed, although not significantly. In the group with adhesive otitis media, an improvement was also seen in each of the tympanograms at 3, 12 and 24 months, although it was not significant in any of them.
